# Healthcare burden among individuals with Angelman syndrome: Findings from the Angelman Syndrome Natural History Study

**DOI:** 10.1002/mgg3.734

**Published:** 2019-05-14

**Authors:** Nasreen Khan, Raquel Cabo, Wen‐Hann Tan, Regina Tayag, Lynne M. Bird

**Affiliations:** ^1^ Ovid Therapeutics Inc. New York New York; ^2^ Division of Genetics & Genomics Boston Children's Hospital, Harvard Medical School Boston Massachusetts; ^3^ PROMETRIKA, LLC Cambridge Massachusetts; ^4^ Clinical Genetics/Dysmorphology University of California, San Diego, Rady Children's Hospital San Diego San Diego California

**Keywords:** healthcare burden, healthcare costs, healthcare resource utilization, medical economics, supportive therapy utilization

## Abstract

**Background:**

The objective of this study is to describe healthcare resource utilization (HRU) and supportive therapy utilization (STU) among individuals with Angelman syndrome (AS), and to compare such usage by molecular etiology.

**Methods:**

Participants were categorized into deletion and non‐deletion genotypes. Statistical differences were assessed using an independent samples *t* test.

**Results:**

Data were available on 302 individuals. Mean age of participants was 5.5 years, 92% of whom were less than 13 years, and 71% had the deletion etiology. About 68% of participants had at least one hospitalization since birth to enrollment in the study; the average number of hospitalizations during that time period was 2.3 and average length of stay was 4.5 days. The most common reasons for hospitalization were seizures, lower respiratory infections, and surgery. The most common reasons for surgery were myringotomy, strabismus surgery, tonsillectomy or adenoidectomy, and gastrostomy tube insertion/fundoplication. Anticonvulsants, gastroesophageal reflux disease, sleep, and behavioral medications were the most commonly prescribed drugs. STU was high among individuals with AS.

**Conclusions:**

This study shows that individuals with AS have high HRU/STU, and apart from a few differences, HRU/STU was similar across molecular etiology. These results reflect usage in younger individuals and studies that describe HRU/STU in older individuals are needed.

## 1 INTRODUCTION

Angelman syndrome (AS) is a rare neurodevelopmental disorder with an estimated prevalence of 1 in 12,000 to 1 in 20,000 people in the United States (NORD, 2018). Individuals with AS present with intellectual disability, severe movement or balance disorder, behavioral issues, seizures, sleep disturbances, and speech impairment (Williams et al., 2006). AS has a significant healthcare burden and requires lifelong support from a network of specialist and caregivers.

AS is caused by loss of *UBE3A* expression in neurons. There are four molecular etiologies: deletion on the maternally inherited copy of chromosome 15q11.2q13.1 (~70% of the cases), paternal uniparental disomy (UPD) for the same chromosomal region (2%–7% of cases), imprinting defect (ID; 3%–5% of cases), and pathogenic variants in the maternal *UBE3A* allele (10% of cases) (Clayton‐Smith & Laan, 2003; Williams et al., 2006). While all cases of AS result in severe global developmental delay and intellectual disability, those with a deletion are more severely affected than those without, and individuals with deletion type are more likely to have microcephaly, poor linear growth, seizures, and more severe motor, cognitive, and communication difficulties (Gentile et al., 2010; Lossie et al., 2001; Moncla et al., 1999; Tan et al., 2011; Varela, Kok, Otto, & Koiffmann, 2004).

Currently, there is no approved treatment for AS and medical management is focused on symptomatic treatment to address the complications of AS and is accompanied by supportive therapies (e.g. physical, occupational, speech, and behavioral therapies). However, there are limited data on the utilization of healthcare resources and supportive therapies in the AS community. This study aims to examine healthcare resource utilization (HRU) and supportive therapy utilization (STU) among individuals with AS and to explore whether there are differences among individuals with AS due to different molecular etiologies. HRU is defined as hospitalization, surgery, and medication use, and STU includes early childhood intervention, as well as physical, occupational, and speech therapy programs. To evaluate both HRU and STU, we used data collected from the AS Natural History Study (ASNHS) baseline visits.

## 2 MATERIAL AND METHODS

### 2.1 Ethics approval

This study was approved by the institutional review boards at each of the institutions where the data were obtained.

### 2.2 Data

The ASNHS, conducted by the Angelman, Rett, and Prader–Willi Syndromes Consortium of the National Institute of Health (NIH) Rare Diseases Clinical Research Network (ClinicalTrials.gov identifier: NCT00296764), was a longitudinal observational study on the developmental progress, behavior, and medical morbidity of individuals with AS (Tan et al., 2011). Individuals were recruited at six study sites across the United States (Rady Children's Hospital San Diego, Texas Children's Hospital, Vanderbilt University Medical Center, Greenwood Genetic Center, Cincinnati Children's Hospital Medical Center, and Boston Children's Hospital) between 2006 and 2014. Inclusion criteria were a molecular diagnosis of AS and participants between the ages of 1 day and 60 years. Individuals were excluded from the study if they had a comorbid disorder that was not a known feature of AS or severe prematurity (less than 28 weeks gestation). A total of 302 individuals with AS were enrolled in this study.

Historical, medical, and resource utilization data were collected from the caregivers of AS individuals since birth until the age of enrollment. HRU and STU data collected included information on hospitalization, reasons for hospitalization, and length of stay (LOS). Surgery data included the date and type of surgery performed. Data were also collected on the use of prescription and non‐prescription medications, including reasons for use. Information on STU included use of early childhood intervention, physical therapy, occupational therapy, and speech therapy. For each STU, information was collected on whether therapy was received, whether it was conducted in school or out of school, and the frequency of therapy.

### 2.3 Statistical analyses

Analyses were performed on the HRU and STU data collected at baseline, which captured utilization from birth until the age of enrollment. To assess differences by molecular etiologies, individuals were stratified into deletion and non‐deletion cohorts. The non‐deletion cohort included UPD, ID, and *UBE3A* mutations. Key outcomes of interest (hospitalization, surgery, medication use, and STU) were summarized for the overall population and by molecular etiology. Other variables of interest, including demographics, medical problems, and clinical seizure history, were summarized for the overall cohort and by molecular etiology. For continuous variables, data were presented as mean, median, standard deviation (*SD*), and 95% confidence interval (CI), as applicable. For categorical variables, data were presented as frequencies and percentages. Statistical differences between the deletion and non‐deletion cohorts were examined using an independent samples *t* test or Fisher's exact test, as appropriate. A nominal *p*‐value of 0.05 or below was considered statistically significant. Missing data were not imputed. All statistical analyses were performed using SAS^®^ Version 9.4 or higher for Windows.

## 3 RESULTS

### 3.1 Descriptive demographic, medical, and seizure history

**Figure 1:**
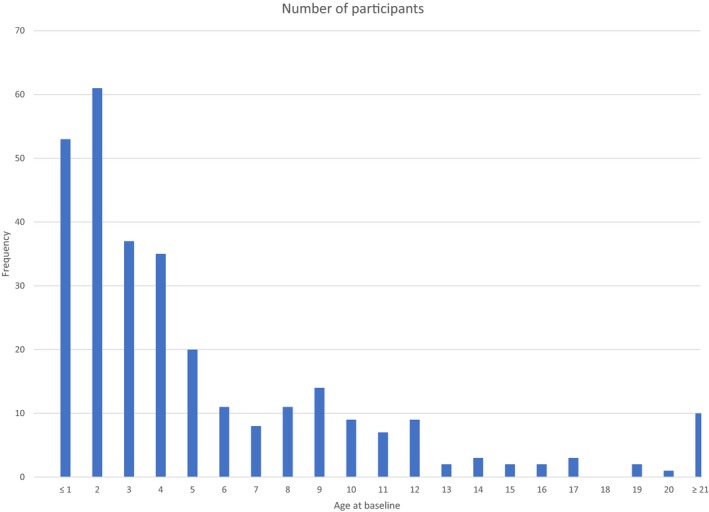
ASNHS, AS Natural History Study. Age distribution at baseline (time of enrollment in the ASNHS study).

Table 1 shows descriptive demographic information, medical problems, and clinical seizure history for the overall cohort and stratified by molecular etiology. Of the 302 participants included in the analyses, three were included only in the overall cohort because it was not documented if they had a deletion. There were 212 participants (71%) with a deletion molecular subtype and 87 participants (29%) without a deletion. Among the non‐deletion etiology, 33 (38%) had a *UBE3A* mutation, 29 (33%) had UPD, 22 (25%) had ID, and 3 (3%) had abnormal DNA methylation without further characterization of the molecular etiology. For the overall cohort, the mean age at AS diagnosis was 2 years (*SD*: 3 years) and about 52% of the participants were female. Mean age at baseline visit was 5.5 years (*SD*: 5.9 years). Figure 1 presents age distribution, 92% of whom were less than 13 years of age; half of the sample was younger than 4 years. A total of 68% of participants had a history of clinical seizures at baseline, and the mean age at first seizure was 1.7 years (*SD* = 1.4 year). Among those who had seizures, the most common types were atonic/drop attacks (42%), tonic/generalized tonic (36%), and absence (35%). The most commonly reported medical issues since birth were sleep issues (82%), gastroesophageal reflux disease (GERD; 66%), otitis media (51%), strabismus (50%), and gagging (50%).

**Table 1:** Descriptive demographic, medical, and seizure history among individuals with AS by molecular etiology

Variable	Overall *N* = 302	Deletion *N* = 212	Non‐deletion *N* = 87	*p*‐value^a^
Molecular diagnosis, *n* (%)				
Abnormal DNA methylation	6 (2)	—	3 (3)	—
*UBE3A* mutation	33 (11)	—	33 (38)	
UPD	29 (10)	—	29 (33)	
Imprinting defect	22 (7)	—	22 (25)	
Deletion	212 (70)	212 (100)	—	
Age at baseline, years, mean (*SD*)	5.5 (5.9)	5.5 (6.5)	5.6 (4.2)	0.9338
Age at diagnosis, years; mean (*SD*)	2 (3)	1.7 (3.1)	2.9 (2.5)	0.0007
Male, *n* (%)	145 (48)	96 (45)	46 (53)	0.2527
Female, *n* (%)	157 (52)	116 (55)	41 (47)	
Seizure history, *n* (%)				
Clinical seizures	199 (68)	156 (77)	42 (49)	<0.0001
Age at first seizure years; mean (*SD*)	1.7 (1.4)	1.5 (1.2)	2.3 (1.6)	0.0025
Seizure type^b^, *n* (%)				
Absence	70 (35)	54 (35)	16 (38)	0.7177
Myoclonic	40 (20)	32 (21)	8 (19)	1.0000
Atonic/drop attacks	83 (42)	61 (40)	21 (50)	0.2203
Tonic/generalized tonic	71 (36)	64 (41)	7 (17)	0.0035
Generalized clonic	46 (23)	42 (27)	4 (10)	0.0223
Other	53 (27)	43 (28)	9 (21)	0.5538
Had ketogenic diet to control seizures	5 (3)	5 (3)	—	
Other medical history, *n* (%)				
Otitis media	149 (51)	98 (48)	50 (59)	0.1210
Pneumonia	72 (25)	50 (25)	22 (27)	0.7650
Gastrointestinal reflux				
Never formally diagnosed	59 (20)	39 (19)	20 (23)	0.1312
Diagnosed	136 (46)	91 (44)	45 (52)	
Vomiting with feeds	61 (21)	43 (21)	17 (20)	0.8751
Gagging	146 (50)	102 (50)	42 (50)	1.0000
Tight heels cords/toe walking	109 (40)	70 (37)	37 (45)	0.2809
Strabismus	147 (50)	116 (57)	31 (37)	0.0028
Sleep issues	241 (82)	168 (81)	71 (85)	0.6129

Note

Percentages have been rounded to the nearest integer.

Abbreviations: AS, Angelman syndrome; *SD*, standard deviation; UPD, uniparental disomy.

a *p*‐values comparing deletion versus non‐deletion cohort were performed using an independent samples *t* test or Fisher's exact test, as appropriate.

b Individuals can be counted in more than one seizure type.

Deletion and non‐deletion individuals had similar characteristics, except for a few notable differences. Specifically, deletion individuals were diagnosed earlier (1.7 years vs. 2.9 years; *p* = 0.0007) and were more likely to have strabismus (57% vs. 37%; *p* = 0.0028). Deletion individuals were also more likely to have clinical seizures (77% vs. 49%; *p* < 0.001) with a mean age at first clinical seizure of 1.5 years compared to 2.3 years for non‐deletion individuals (*p* = 0.0025). In addition, individuals with deletion were more likely to have tonic/generalized tonic (*p* = 0.0035) and generalized clonic (*p* = 0.0223) seizures compared to non‐deletion individuals.

### 3.2 Healthcare resource utilization

Tables 2 and 3 present the HRU for the AS cohort overall and stratified by molecular etiology. Sixty‐eight per cent of participants had at least one hospitalization since birth to enrollment into the study; the mean number of hospitalizations during that time period was 2.3 (95% CI: 2.1–2.5). The average LOS was 4.5 days (95% CI: 3.8–5.2). The most common reasons for hospitalization were seizures (40%), lower respiratory infections (21%), and surgery (11%; Table 3). Deletion and non‐deletion individuals had similar rates of hospitalization, number of hospitalizations, and LOS. Reasons for hospitalization were similar across molecular etiologies except for seizures, which was significantly more common among the deletion cohort at 48% compared to the non‐deletion cohort at 21% (*p* = 0.0004; Table 3).

**Table 2:** HRU and STU for overall cohort and by molecular etiology

Descriptive	Overall *N* = 302	Deletion *N* = 212	Non‐deletion *N* = 87	*p*‐value^a^
HRU				
Hospitalization				
Ever been hospitalized, *n* (%)	205 (68)	146 (69)	57 (66)	0.5875
Number of hospitalization, mean (95% CI)	2.3 (2.1–2.5)	2.3 (2.0–2.5)	2.4 (2–2.9)	0.7099
Length of stay, mean (95% CI)	4.5 (3.8–5.2)	4.1 (3.4–4.9)	5.5 (3.9–7.2)	0.1300
Surgery				
Ever had surgery, *n* (%)	172 (57)	119 (56)	51 (59)	0.7022
Number of surgeries, mean (95% CI)	1.7 (1.6–1.8)	1.7 (1.5–1.9)	1.7 (1.5–1.9)	1.0000
Medication use				
Ever used prescription medications, *n* (%)	245 (81)	181 (85)	62 (71)	0.0084
Number of prescription medications, mean (*SD*)	2.8 (2.0)	2.7 (1.8)	3.2 (2.3)	0.1242
STU				
Early childhood intervention, *n* (%)	279 (95)	196 (94)	81 (96)	1.0000
Physical therapy, *n* (%)	263 (90)	190 (92)	71 (86)	0.0837
Occupational therapy, *n* (%)	255 (88)	181 (87)	72 (88)	0.5981
Speech therapy, *n* (%)	251 (86)	175 (85)	74 (89)	0.7332

Abbreviations: CI, confidence interval; HRU, healthcare resource utilization; STU, supportive therapy utilization.

a *p*‐values comparing deletion versus non‐deletion cohort were performed using an independent samples *t* test or Fisher's exact test, as appropriate.

Includes prescription medications used by more than 10 individuals.

**Table 3:** Most frequent reasons for HRU for overall cohort and by molecular etiology

Descriptive	Overall *N* = 302	Deletion *N* = 212	Non‐deletion *N* = 87	*p*‐value^a^
Hospitalization reasons, *n* (%)				
Seizure	83 (40)	70 (48)	12 (21)	0.0004
Lower respiratory infection	43 (21)	28 (19)	15 (26)	0.3390
Surgery	22 (11)	17 (12)	4 (7)	0.4449
Other infection	18 (9)	11 (8)	7 (12)	0.2836
Dehydration	16 (8)	12 (8)	4 (7)	1.0000
Tonsillectomy and adenoidectomy	13 (6)	7 (5)	6 (11)	0.1978
Surgery reasons, *n* (%)				
Myringotomy	59 (34)	39 (33)	20 (39)	0.4827
Strabismus	52 (30)	43 (36)	9 (18)	0.0184
Tonsillectomy or adenoidectomy	43 (25)	25 (21)	17 (33)	0.1197
G‐tube or fundoplication	14 (8)	8 (7)	6 (12)	0.3604
Inguinal hernia	10 (6)	7 (6)	2 (4)	0.7257
Medication reasons				
AED, *n* (%)				
Clonazepam	72 (24)	55 (26)	16 (18)	0.1805
Levetiracetam	67 (22)	55 (26)	11 (13)	0.0136
Valproic acid	66 (22)	49 (23)	17 (20)	0.5423
Topiramate	48 (16)	38 (18)	9 (10)	0.1170
Lamotrigine	39 (13)	28 (13)	11 (13)	1.0000
Phenobarbital	32 (11)	25 (12)	7 (8)	0.4137
Diazepam	19 (6)	18 (8)	1 (1)	0.0173
GERD, *n* (%)				
Ranitidine	72 (24)	44 (21)	27 (31)	0.0722
Lansoprazole	56 (19)	39 (18)	17 (20)	0.8706
Metoclopramide	35 (12)	24 (11)	11 (13)	0.8432
Omeprazole	28 (10)	17 (8)	11 (13)	0.2734
Sleep/behavior, *n* (%)				
Melatonin (OTC)	126 (42)	89 (42)	36 (41)	1.0000
Clonidine	41 (14)	23 (11)	17 (20)	0.0603
Diphenhydramine (OTC)	24 (8)	17 (8)	11 (13)	0.3543
Risperidone	20 (7)	11 (5)	9 (10)	0.1265
Constipation/laxative, *n* (%)				
Polyethylene glycol	46 (15)	32 (15)	13 (15)	1.0000
Laxative (others)	17 (6)	13 (6)	4 (5)	0.7855

Abbreviation: AED, Antiepileptic drugs; GERD, gastroesophageal reflux disease; OTC: Over‐the‐counter.

a *p*‐values comparing deletion versus non‐deletion cohort were performed using an independent samples *t* test or Fisher's exact test, as appropriate.

Fifty‐seven per cent of participants with AS had at least one surgery (Table 2). The most common surgeries were insertion of ear tubes (34%), correction of strabismus (30%), tonsillectomy and adenoidectomy (25%), and gastrostomy tube insertion/fundoplication (8%; Table 3). Mean number of surgeries since birth to time of enrollment in the study was 1.7 (95% CI: 1.6–1.8). Deletion and non‐deletion individuals had similar rates, mean number, and reasons for surgery except for strabismus, which was significantly more common in the deletion cohort compared to the non‐deletion cohort (36% vs. 18%; *p* = 0.0184).

About 80% of participants used at least one prescription medication (Table 2). The mean number of prescription medications used was 2.8 (*SD*: 2.0). Anticonvulsants were the most common prescription medications that were used, with clonazepam (24%), levetiracetam (22%), valproic acid (22%), and topiramate (16%) being the most common. This was followed by the use of medications for GERD: ranitidine (24%) and lansoprazole (19%) and medications for sleep or behavior: melatonin (42%), clonidine (14%), diphenhydramine (8%), and risperidone (7%) (Table 3). Prescription medication use was higher among the deletion cohort compared to the non‐deletion group (85% vs. 71%; *p* = 0.0084). However, specific medications did not differ between cohorts (Table 3) except for levetiracetam, which was used significantly more in the deletion cohort (*p* = 0.0136).

### 3.3 Supportive therapy utilization

Table 2 also presents STU among individuals with AS. In general, the use of therapy was high, with 95% of individuals receiving early child intervention, 90% receiving physical therapy, 88% receiving occupational therapy, and 86% receiving speech therapy (either school‐ or non‐school‐based). STU did not differ between the deletion and non‐deletion cohorts.

## 4 DISCUSSION

To our knowledge, this is the first published study to systematically characterize the healthcare burden of AS in the United States of America (USA). This is also the first published study to analyze the healthcare burden among individuals with AS by molecular etiology. A targeted review of the literature identified only two published studies that have assessed healthcare utilization among individuals with AS (Domínguez‐Berjón, Zoni, Esteban‐Vasallo, Sendra‐Gutierrez, & Astray‐Mochales, 2018; Thomson, Glasson, & Bittles, 2006). Domínguez‐Berjón et al. (2018) examined 49 individuals with AS in Spain with a median age of 17 years (interquartile range 14); however, only 32 were confirmed to have AS by molecular testing. Thomson et al. (2006) studied 34 individuals with AS in Western Australia with a mean age of 22 years (range 6.5–39 years), but only 14 were confirmed to have AS by molecular testing. These two studies had relatively few individuals with genetically confirmed AS and described only the incidence and reasons for hospitalizations without analyzing the differences among individuals with different molecular etiologies. As such, our study makes a significant contribution by characterizing the current medical and therapeutic needs of a relatively large sample of individuals with genetically confirmed AS in the USA.

This study suggests that individuals with AS experience significant healthcare burden. Individuals with AS are known to experience seizures, motor, speech, behavior, and cognition issues, and this was reflected in the vast array of medical services and supportive therapy used in our sample. More than half of the sample had at least one episode of hospitalization. These results are consistent with the two other AS studies that examined healthcare resource utilization. Domínguez‐Berjón et al. (2018) found that 86% of individuals with AS had at least one hospitalization. Major causes of hospitalization in our study were seizures, lower respiratory infection (e.g. pneumonia, bronchitis), or surgery. The commonly reported reasons for hospitalization in the Domínguez‐Berjón et al. (2018) study were oral‐dental care, seizures, orthopedic problems, and acute respiratory disorders. Thomson et al. (2006) reported a median number of hospitalizations of 5.5 per person and the most common reasons for hospitalization in their cohort were epilepsy, gastrointestinal disorders, and dental issues. The magnitude of hospitalization was different between this study and the Spanish study. Specifically, we found a lower rate of overall hospitalization (68% vs. 86%) but a higher rate of seizure‐related hospitalizations (40% vs. 20%) compared to the Spanish study. This could be because we did not consider outpatient dental cleanings or restorations as “hospitalization”, or it could reflect differences in the healthcare practices between the two countries. Additionally, the individuals in our cohort are younger (median age: 3 years) compared to those in the Spanish study (median age: 17 years) which could also explain differences in hospitalization rates.

We also found that use of prescription and non‐prescription medication was high among individuals with AS, with the types of medications reflecting the major medical problems experienced (e.g. sleep disorders, seizures, gastrointestinal problems). The type of seizure medications used in this study were similar to those reported in published literature (Fiumara, Pittala, Cocuzza, & Sorge, 2010; Williams et al., 2006). We observed a significant use of melatonin and diphenhydramine in this study, suggesting that sleep disorders are a major issue in this population.

This is the first published study to report STU among individuals with AS. Approximately, 86%–96% of the participants in our study had physical, occupational, and/or speech therapy, suggesting the profound impact that AS has on an individual's global functioning.

The secondary objective of this study was to assess whether there were differences in healthcare utilization across individuals with deletion and non‐deletion etiology. Previous studies suggested that individuals with deletion experienced higher rates of seizures, feeding difficulties, microcephaly, and pronounced motor, cognitive, and communication difficulties (Lossie et al., 2001; Moncla et al., 1999; Varela et al., 2004). In our sample, the deletion cohort was significantly more likely to have seizures, and their seizures started at a younger age. However, other clinical characteristics were comparable across groups. Notably, HRU and STU between the deletion and non‐deletion cohorts were similar except for seizure‐related hospitalization, strabismus surgery, and use of levetiracetam. Hence, despite suggestions in the literature of a slightly more severe phenotype, this analysis demonstrates that the healthcare burden across the two etiologies is comparable. Future studies that investigate how this burden varies with age would help assess if healthcare needs remain similar across the two etiologies over time.

This study has several limitations. This was an observational study that relied upon caregiver recall of healthcare utilization from birth to the time of the natural history study visit; therefore, actual healthcare burden may be underrepresented. Another limitation is that most of the individuals in this study (92%) were under the age of 13 years, which inevitably underestimates the true burden of healthcare utilization over the lifetime of an individual with AS. The primary reason for the predominance of younger children in our cohort was that co‐enrollment in other treatment trials was restricted to participants of younger ages. Hence, the utilization reported in this study is less reflective of the needs of adults and adolescents. Similarly, the medical and seizure history is more reflective of younger individuals. For instance, we found that only 68% of the entire cohort had a history of clinical seizures. This is in contrast to the published clinical diagnostic criteria for AS, which states that seizures are observed in about 80% of AS individuals (Williams et al., 2006). The lower history of clinical seizure is mainly because the population is skewed toward younger individuals and seizures just may not have happened yet. Indeed, when data were examined by age, history of clinical seizures among 5 years and older was 80% and among 13 years and older was 100%. The younger age can also explain differences in hospitalization rates between ours and other published studies as well as relatively similar utilization across the two etiologies. Hence, future studies that analyze HRU and STU by age and that include older individuals are needed to assess changes in burden with age and to assess unmet needs among older individuals. Finally, the ASNHS did not capture all of the resources used by the individuals with AS such as outpatient visits or use of communication devices (e.g. augmentative and alternative communication, iPad^™^), which have become increasingly more prevalent to aid in communication (Wheeler, Sacco, & Cabo, 2017).

In conclusion, this is the first known published study to report HRU and STU among children with AS in the USA. The high rates of HRU and STU by individuals with AS highlight the significant healthcare burden caused by this disorder in this age group. In addition, this is the first study to show that the healthcare burden was similar across the molecular etiologies among younger individuals with AS. However, additional studies need to be performed to understand how healthcare burden varies with age and among older individuals with AS to further evaluate the impact of AS on a broader population and the healthcare system.

## CONFLICT OF INTEREST

Dr. Khan is a paid consultant for Ovid Therapeutics. Ms. Cabo is a paid employee of Ovid Therapeutics. Dr. Tan is participating in clinical trials supported by Ovid Therapeutics and Dimension Therapeutics, and has also received research support from Ovid Therapeutics. Ms. Tayag is a paid employee of PROMETRIKA, LLC. PROMETRIKA was paid by Ovid Therapeutics to perform statistical analyses for this paper. Dr. Bird is participating in clinical trials supported by Ovid Therapeutics.
